# Continuous DNA replication is required for late gene transcription and maintenance of replication compartments in gammaherpesviruses

**DOI:** 10.1371/journal.ppat.1007070

**Published:** 2018-05-29

**Authors:** Dajiang Li, Wenmin Fu, Sankar Swaminathan

**Affiliations:** 1 Division of Infectious Diseases, Department of Medicine, University of Utah School of Medicine, Salt Lake City, Utah, United States of America; 2 Department of Medicine, George E. Wahlen Veterans Affairs Medical Center, Salt Lake City, Utah, United States of America; University of Pennsylvania Medical School, UNITED STATES

## Abstract

Late gene transcription in herpesviruses is dependent on viral DNA replication *in cis* but the mechanistic basis for this linkage remains unknown. DNA replication results in demethylated DNA, topological changes, removal of proteins and recruitment of proteins to promoters. One or more of these effects of DNA replication may facilitate late gene transcription. Using 5-azacytidine to promote demethylation of DNA, we demonstrate that late gene transcription cannot be rescued by DNA demethylation. Late gene transcription precedes significant increases in DNA copy number, indicating that increased template numbers also do not contribute to the linkage between replication and late gene transcription. By using serial, timed blockade of DNA replication and measurement of late gene mRNA accumulation, we demonstrate that late gene transcription requires ongoing DNA replication. Consistent with these findings, blocking DNA replication led to dissolution of DNA replication complexes which also contain RNA polymerase II and BGLF4, an EBV protein required for transcription of several late genes. These data indicate that ongoing DNA replication maintains integrity of a replication-transcription complex which is required for recruitment and retention of factors necessary for late gene transcription.

## Author summary

Herpesviruses exhibit both latent and lytic replication cycles. Gammaherpesviruses such as Kaposi’s sarcoma-associated herpesvirus and Epstein Barr virus undergo lytic replication when they reactivate from latency. During this process, when infectious virions are produced, an orderly cascade of gene expression occurs. Late lytic genes, which primarily encode structural components of the virion, are only transcribed after replication of the DNA genome has occurred. Unlike early lytic genes, late gene transcription is tightly linked to viral DNA replication; if viral DNA replication is blocked, late gene mRNA accumulation is severely inhibited. The mechanism by which late gene transcription is linked to DNA replication has remained elusive. In this paper we show that a process of continuous DNA replication is required. If one blocks DNA replication, further transcription also ceases, indicating that concurrent DNA replication is required to maintain late transcription. We also show that when DNA replication is blocked, the nuclear complexes in which herpesviruses are replicating dissociate. These replication complexes also serve as factories of viral transcription. When the complexes disperse, proteins required for transcription dissociate from the DNA replication machinery. These data indicate that ongoing DNA replication is necessary to maintain the physical and functional integrity of these structures. Our study provides new insight into this linkage that ensures coordination between viral replication and late gene expression.

## Introduction

The human gammaherpesviruses Kaposi's sarcoma-associated herpesvirus (KSHV) and Epstein-Barr virus (EBV) establish lifelong persistent infection in B lymphocytes and intermittently reactivate, producing infectious virions transmitted by the oral route [1, 2]. KSHV is associated with Kaposi’s sarcoma, Multicentric Castleman’s disease and primary effusion lymphoma, whereas EBV is linked to nasopharyngeal carcinoma and a variety of lymphoproliferative syndromes and lymphomas [1, 2]. In common with all herpesviruses, both KSHV and EBV sequentially express immediate-early (IE), early (E) and late (L) genes during the lytic phase of replication. Immediate-early genes in the γ-herpesviruses encode transcriptional activators that initiate the lytic cycle and are necessary for virus reactivation from latency [3]. Many early gene products are required for lytic viral DNA replication, which is carried out by virus-encoded homologs of the cellular DNA replication machinery [3]. Late genes encode the structural virion components that comprise capsid, tegument and glycoproteins [1]. In addition to these essential aspects of virion production, lytic gene products are increasingly recognized as contributing to tumorigenesis [4]. Intermittent lytic replication and virion production may also contribute to maintenance of the latently infected reservoir in vivo [4]. Understanding the molecular mechanisms by which sequential gene expression is controlled during the lytic phase of replication is therefore important for identifying molecules and pathways that can be therapeutically targeted.

Whereas expression of IE and E genes is unaffected by inhibition of DNA replication [5], most EBV and KSHV late genes are strictly dependent on DNA replication from the lytic origin of replication, and inhibitors of viral DNA polymerase drastically inhibit late gene expression [6, 7]. The basis for lytic DNA replication-dependent late gene expression has been a subject of investigation for many years but remains incompletely characterized. Whether DNA replication from an origin *in cis* to the late gene promoter is required for late gene transcription has been controversial. Two studies in EBV indicated that late promoter driven gene expression from plasmids could occur in the absence of plasmid DNA replication [8, 9]. However, several studies using plasmid reporters and late promoters have concluded that the presence of DNA replication *in cis* is necessary to allow efficient late gene expression in KSHV, MHV-68 and EBV although virally produced proteins acting *in trans* are also necessary [10–13]. Similar conclusions were also drawn regarding HSV late gene expression from plasmids and from the viral genome [14, 15].

Recent findings have revealed fundamental differences between late promoters and IE, E and cellular promoters in β and γ herpesviruses that provide a starting point for understanding the regulatory mechanisms of late genes in β and γ herpesviruses [8, 13, 16–21]. KSHV, EBV, murine gammaherpesvirus 68 and human cytomegalovirus all express a complex of six proteins which form a viral pre-initiation complex (vPIC) specific for late gene promoters and which are essential for late promoter function [8, 16–21]. Homologous genes have also been identified in human HHV6 [22]. One of the proteins in the EBV vPIC, the BcRF1 gene product, functionally substitutes for TBP and binds to atypical TATT boxes in late promoters [18]. The vPIC has been demonstrated to bind RNAP at late promoters at which the vPIC forms [8, 18]. A recent elegant study used recombinant EBV bacmids to demonstrate that each of the six components is individually required for late gene expression but do not affect DNA replication [23]. Further, this study confirmed that late gene expression from the EBV genome requires DNA replication *in cis*. Thus, it appears that the unique virus-specified late gene transcriptional machinery is dependent on DNA replication of the template on which the late gene promoter resides.

Despite the discovery of the specific vPIC involved in β and γ herpesvirus late gene expression, the basis of its unique dependence on DNA replication *in cis* remains to be explained. Several possible mechanisms, which are not mutually exclusive, may be considered. First, late gene transcription may require chromatin modifications that occur concurrent with lytic genome replication, such as removal of methylation marks and histone occupancy [24, 25]. The “naked” chromatin of newly replicated genomes may thereby allow more efficient access of the vPIC to late gene promoters. Alternatively, the translocating DNA replication complex may bring or recruit factors or components of the vPIC required for late gene transcription to the relevant promoters. Third, the physical localization of replicating genomes may facilitate or be required for co-localization of transcription factors needed for late gene transcription. For example, EBV lytic DNA replication occurs at discrete sites, called replication compartments, in which viral transcription also takes place. These include domains designated BMRF1 cores, in which newly synthesized viral DNA genomes are organized around and then stored inside cores composed of BMRF1, the DNA polymerase processivity factor [26]. It has also recently been shown that KSHV assembles an "all-in one" factory for both gene transcription and DNA replication [27]. Finally, DNA replication increases template copy number and may contribute to late transcript abundance. In this study, we have investigated the involvement of several of these possible mechanisms, including demethylation, template abundance and the role of ongoing DNA replication and replication compartment formation. Our findings suggest that demethylation and template abundance are not significant factors in the linkage between late gene expression and DNA replication in KSHV and EBV. However, continuous, ongoing DNA replication appears to be necessary for late gene expression and maintenance of replication compartments.

## Results

### DNA demethylation enhances early and late gene expression but cannot rescue late gene expression when DNA replication is inhibited

**Fig 1:**
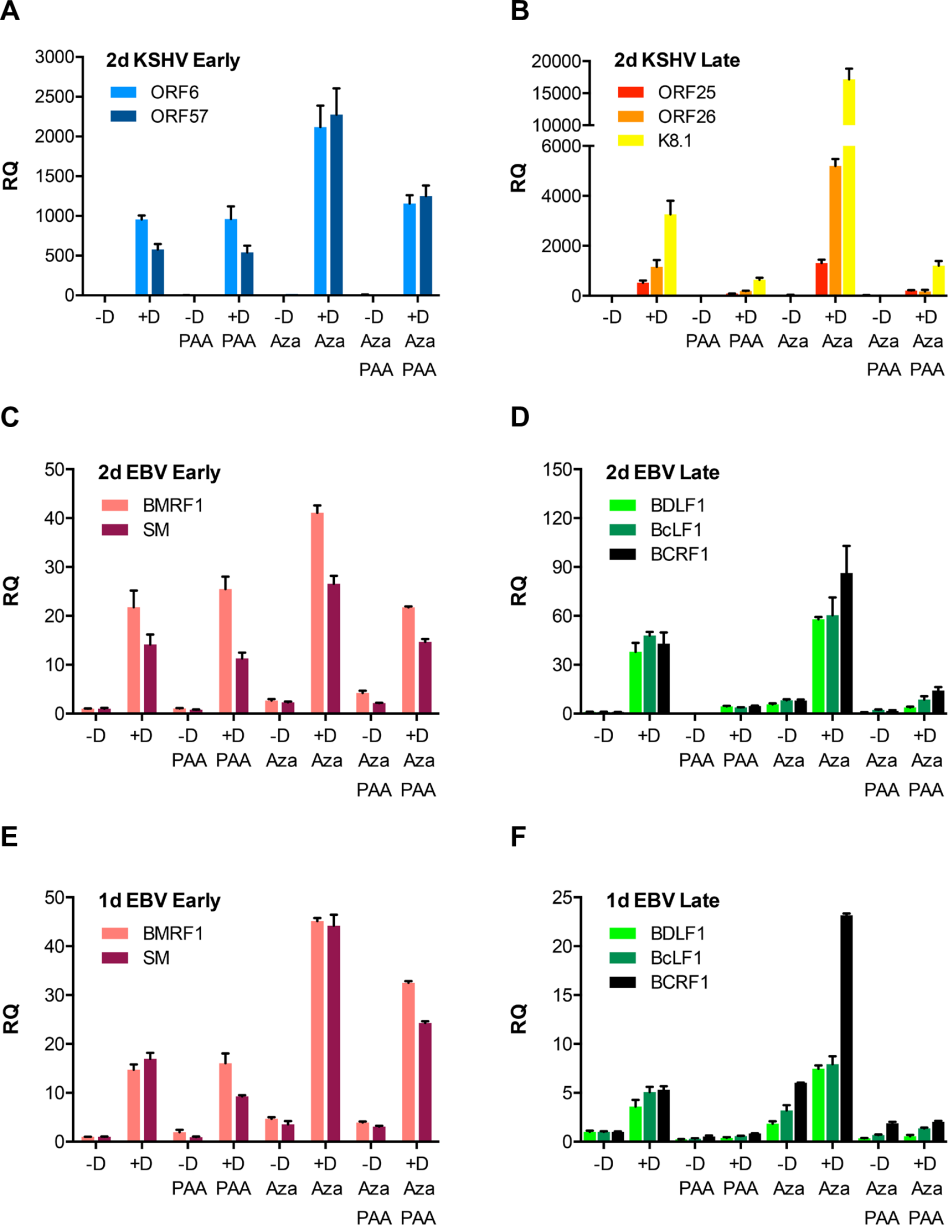
Effect of 5-Aza and PAA on KSHV and EBV lytic gene expression. RNA was harvested from iSLK cells (KSHV) or AGSiZ cells (EBV) after doxycycline induction of lytic replication (+D) or mock induction (-D). Cells were also treated with 5-Aza or PAA as indicated. qPCR was performed to measure relative quantity (RQ) of each mRNA. A. KSHV lytic early genes [ORF6, ORF57] at 48h post-induction. B. KSHV late genes (ORF25, ORF26 or K8.1] at 48h. C. EBV early genes [BMRF1, SM] at 48h. D. EBV late genes (BDLF1, BcLF1 and BCRF1) at 48h. E. EBV early genes at 24h. F. EBV late genes at 24h. Error bars represent SEM.

Early experiments demonstrated that promoter hypomethylation correlated with transcriptional activity of EBV lytic genes but that demethylation alone was insufficient to induce lytic gene expression [28, 29]. It is also known that DNA hypomethylation occurs during lytic replication and that both EBV and KSHV virion DNA are essentially devoid of DNA methylation and histones [25, 29]. It was therefore possible that DNA hypomethylation that occurs coincident with lytic replication and production of linear DNA templates might be required for efficient transcription of late genes. In order to determine whether demethylation of KSHV latent DNA could rescue late gene transcription when DNA replication was blocked, we used the demethylase inhibitor 5-azacytidine (5-Aza) in combination with phosphonoacetate (PAA) a selective inhibitor of herpesvirus DNA polymerases [30]. BAC16/iSLK cells are epithelial cells that inducibly express RTA (ORF50) upon treatment with doxycycline and stably carry the eGFP-expressing JSC-1 KSHV bacmid BAC16 [31, 32]. RTA expression is necessary and sufficient to induce lytic KSHV replication [33], and treatment of BAC16/iSLK with doxycycline leads to highly efficient induction of the KSHV lytic cycle and production of infectious KSHV [19]. BAC 16/iSLK cells were cultivated in media supplemented with 2μM 5-Aza for two days to demethylate DNA before induction of lytic replication. Lytic viral replication was induced with doxycycline and DNA replication was simultaneously inhibited with PAA. Two days post induction, RNA and DNA were harvested and qPCR was performed to quantitate individual gene expression and viral genome copy number. As expected, RNA transcription of early genes (ORF6 and ORF57) was highly induced by doxycycline but was unaffected by PAA treatment (Fig 1A). There was a further increase when iSLK cells were treated with 5-Aza, indicating that methylation inhibits early gene promoter activity. Demethylation also upregulated expression of late genes, (ORF25, ORF26 and K8.1, Fig 1B). However, consistent with the dependence of late gene transcription on DNA replication, PAA completely inhibited transcription of K8.1, ORF25 and ORF26. Importantly, even when viral genomes were demethylated by 5-Aza, transcription was strongly inhibited by PAA, demonstrating that removal of methylation is not the reason that DNA replication is required for late lytic gene transcription.

In order to determine whether the dependence of late gene transcription on DNA replication was similarly independent of the methylation status of template DNA during EBV replication, we repeated the same experiment in AGSiZ cells. AGSiZ is an EBV bacmid infected gastric carcinoma-derived cell line in which a doxycycline-inducible EBV transactivator Zta has been stably introduced by lentivirus transduction [34]. As expected, EBV early genes (BMRF1 and SM) were highly expressed without being inhibited by PAA treatment (Fig 1C) and a further increase in BMRF1 and SM mRNA levels was observed upon treatment with 5-Aza. Expression of late genes (BDLF1, BcLF1 and BCRF1) was highly inhibited by PAA treatment (Fig 1D). However, demethylation did not affect the dependence of late gene transcription on DNA replication as PAA was equally effective in inhibiting late gene expression despite the presence of 5-Aza. These data therefore indicate that DNA demethylation occurring during DNA replication is not the basis of the linkage between late transcription and genome replication in either KSHV or EBV.

**Fig 2:**
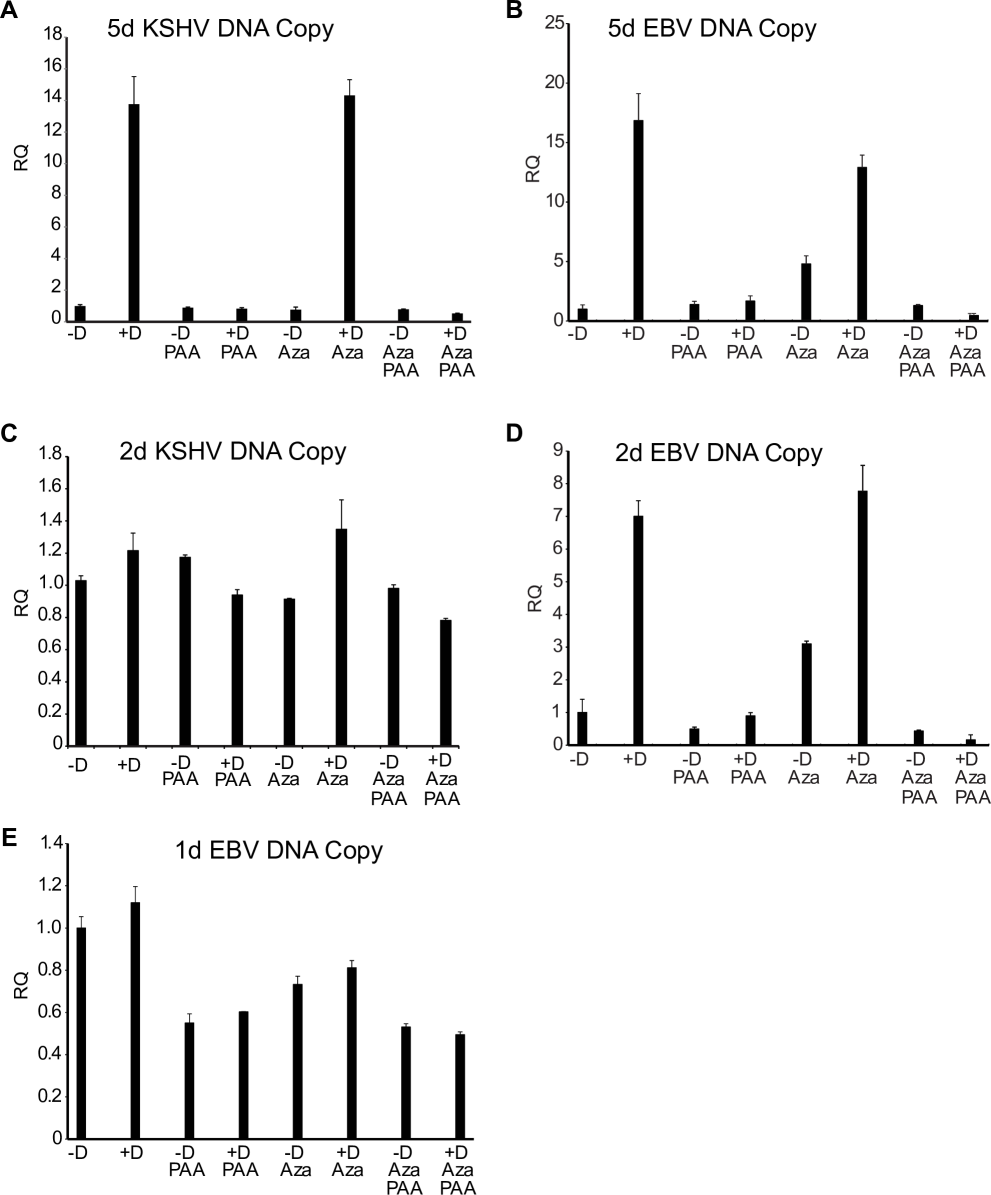
KSHV and EBV DNA accumulation at early and late times after induction of replication. KSHV or EBV lytic replication was induced in iSLK cells or AGSiZ cells respectively. Cells were treated with doxycycline (+D) or mock induced (-D) and either treated or mock-treated with PAA as shown. Samples were also treated with 5-Aza in parallel (Aza). DNA was isolated from cell pellets at times shown and relative quantities of DNA (RQ) were measured by qPCR. A. KSHV genome DNA at 5 days after lytic induction. B. EBV genome DNA at 5 days after lytic induction. C. KSHV genome DNA at 2 days after lytic induction. D. EBV genome DNA at 2 days after lytic induction. E. EBV genome DNA at 1 day after lytic induction. Error bars represent SEM.

We confirmed that PAA had effectively inhibited KSHV and EBV lytic replication by measuring the KSHV and EBV copy number in cells used for the above experiment. Increases in intracellular EBV and KSHV DNA are maximal by 4–5 days post induction. As shown in Fig 2A and 2B, PAA completely blocked the increase in KSHV and EBV genome copy number seen 5 days after induction of lytic replication.

### Late gene transcription is not due to amplification of viral DNA genomes and increased template number

Another possible basis for replication dependent late gene transcription is the increase in template number that occurs with lytic DNA replication. To ask whether there was a significant increase in KSHV template number at 48 hours when 500–1,000-fold increases in late transcript abundance were observed (Fig 1), we measured KSHV DNA copy number at 48 hours after induction. KSHV genome copy number was measured by qPCR of DNA extracted from the same cells that were used for measurement of mRNA transcript levels. As shown in Fig 2C, DNA copy number had only increased by 20% at 48 hours, in the absence of PAA, demonstrating that the large increases in late gene transcript levels precede those in DNA copy number. Increased KSHV template numbers are therefore unlikely to play a role in replication dependent increases in KSHV late gene transcription. When similar measurements were performed to measure EBV copy numbers in AGSiZ cells, the kinetics were different, EBV replication occurring somewhat earlier, with increases in copy number at 48 hr (approximately 7-fold) (Fig 2D). We therefore repeated the experiment in AGSiZ cells and harvested cells for RNA and DNA 24 hr post induction to determine whether late gene transcription could be demonstrated to precede increases in genome copy number. Although as expected, late gene transcription was not as pronounced as at 48 hr, all patterns at 24 hr were similar to those observed at 48 hr: Early gene transcription was insensitive to PAA (Fig 1E) and 5-Aza treatment did not rescue late gene transcription when DNA replication was inhibited (Fig 1F). At this time point, DNA copy number however, had not increased more than 10% from levels prior to induction (Fig 2E). Therefore, increases in template numbers did not play a role in replication dependent stimulation of EBV or KSHV late gene transcription.

### 5-Aza demethylates both cell and viral genome DNA

**Fig 3:**
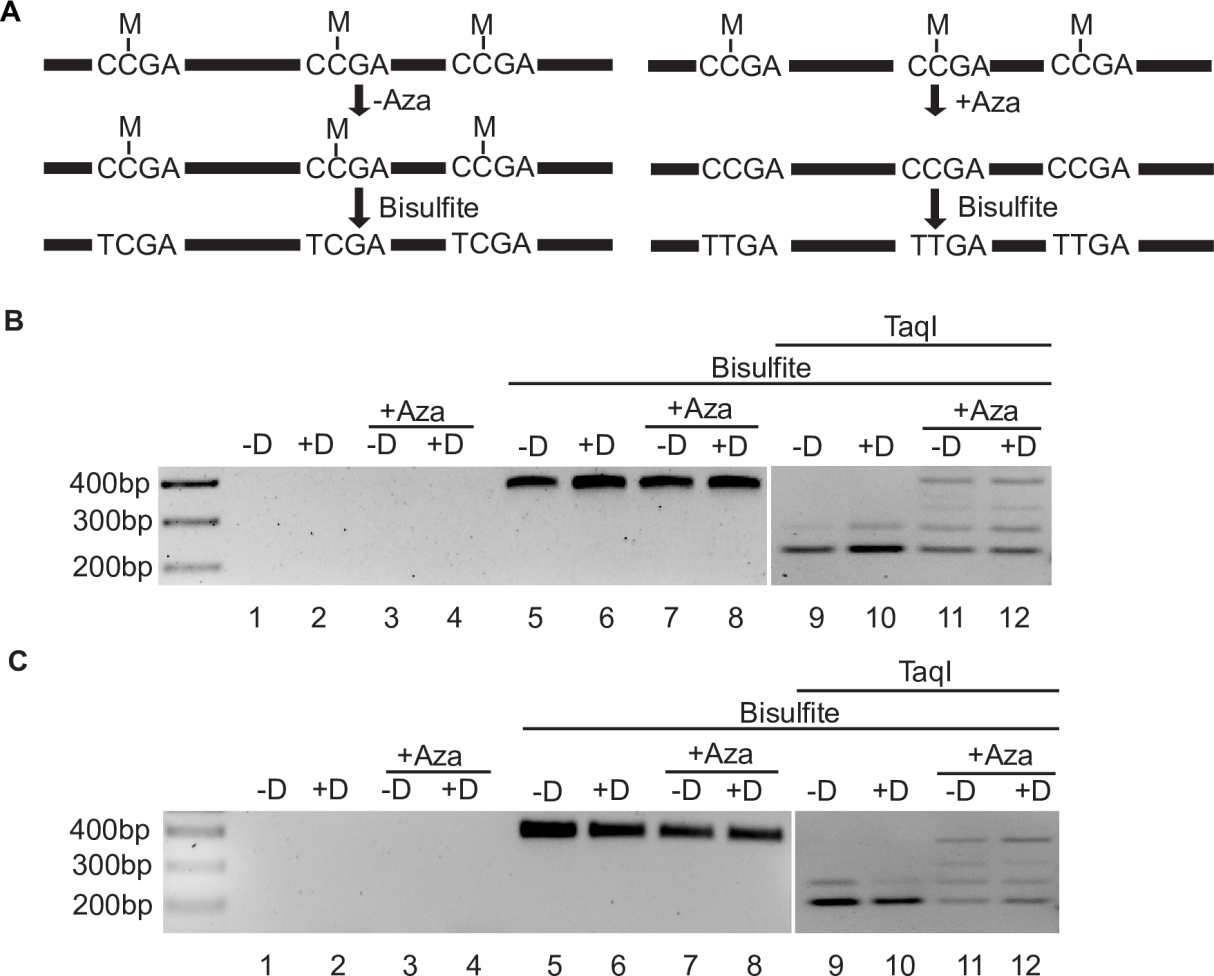
COBRA analysis demonstrating 5-Aza effect on DNA demethylation in iSLK and AGSiZ cells. A. Schematic representation of COBRA. Following bisulfite treatment, CpG methylated sequences are converted from C^m^CGA to T^m^CGA (TaqI sites) in 5-Aza-untreated samples (left panel) while demethylated CCGA are converted to TTGA (losing TaqI sites) in 5-Aza-treated samples (right panel). B. Specific amplification of bisulfite converted DNA from iSLK cells and demonstration of demethylation after 5-Aza treatment. Primers are specific for predicted C to T converted sequences. PCR products from doxycycline-induced (+D) or mock induced (-D) iSLK samples with or without 5-Aza treatment are shown in lanes 1–4, respectively. Bisulfite-converted PCR products from doxycycline-induced (+D) or mock induced (-D) iSLK samples with or without 5-Aza treatment are shown in lanes 5–8, respectively. The amplicon size is 392bp. After digestion of samples with TaqI, 5-Aza untreated samples are almost fully digested to a 211 bp fragment with a minor partial cleavage product at 291 bp (lanes 9, 10). After 5-Aza treatment, TaqI digestion is inhibited due to conversion of unmethylated CCGA to TTGA, and multiple partially cleaved 211 bp, 291 bp, 321 bp and uncleaved (392bp) products are produced (lanes 11, 12). C. Specific amplification of bisulfite converted DNA from AGSiZ cells and demonstration of demethylation after 5-Aza treatment. AGSiZ cells were treated and COBRA results are presented as in Fig 3B.

Although the increases in both early and late gene transcription seen with 5-Aza were consistent with promoter demethylation, we performed direct analyses to confirm CpG demethylation by 5-Aza. We analyzed a known methylated region in the cellular genome (LINE-1, L1-PKP4 [35]) by COBRA (combined bisulfite restriction analysis) (Fig 3). DNA was amplified with primers specific for bisulfite converted DNA (in which unmethylated, but not methylated C, is converted to T) and then digested with TaqI. As shown in Fig 3B and 3C, specific amplification in bisulfite treated samples yielded a 392 bp PCR product, but not in bisulfite untreated samples from iSLK and AGSiZ cells. Bisulfite treatment is predicted to create 3 TaqI cut sites (TCGA) in the PKP4 PCR product, but only from methylated CCGAs (Fig 3A, left panel). Bisulfite-treated PCR products demonstrated almost complete TaqI cleavage, confirming the methylated status of the amplified region (Fig 3B and 3C, lanes 9 and 10). In 5-Aza-treated samples, analysis of the PCR products revealed a mixture of partially cleaved and un-cleaved bands (Fig 3B and 3C, lanes 11 and 12). These data therefore demonstrate that 5-Aza led to CpG demethylation and prevented production of TaqI sites from methylated CCGA sites.

In addition to demethylation of cellular DNA, we wished to confirm that viral genomes were also demethylated by 5-Aza. The methylation status of the ORF33 promoter in KSHV genome, which has been previously demonstrated to be methylated [25], was therefore assessed by pyrosequencing. Five CpG sites in the ORF33 promoter showed changes in the level of methylation upon 5-Aza treatment (Table 1). At 2 days post induction of lytic replication (4 days after 5-Aza treatment), the average percentage change of methylation had decreased in all 5-Aza treated samples. Lytic replication led to demethylation (15.7% change) and when lytic replication was blocked by PAA there was no change in methylation. However, 5-Aza treatment in the presence of PAA led to a degree of demethylation similar to that of lytic replication (15.7% change). This experiment demonstrated that the KSHV viral genome DNA was demethylated by 5-Aza treatment, consistent with its observed effects on gene expression (Fig 1).

**Table 1:** Methylation changes across all CpG in KSHV ORF33 promoter ^a^.

	Percentage average of methylation status	Percentage average change in methylation status
-Dox	10.2%	
+Dox	8.6%	-15.7%
5-Aza - Dox	7.8%	-23.5%
5-Aza + Dox	5.6%	-45%
PAA + Dox	10.4%	+2%
5-Aza + PAA +Dox	8.6%	-15.7%

^a^The table shows the results of pyrosequencing DNA from Bac16 KSHV infected iSLK cells. Cells were treated with 5-Aza or mock-treated as shown. 48 h later, cells were treated with doxycycline to induce lytic replication (+Dox) or mock-induced (-Dox) +/- PAA as shown. DNA was isolated 48 hr post-induction, bisulfite converted and analyzed by pyrosequencing. The average percentage methylation at the five CpG sites in the promoter is shown. Sequencing data is provided in S1 Fig.

### KSHV and EBV late gene transcription depend on ongoing DNA replication

**Fig 4:**
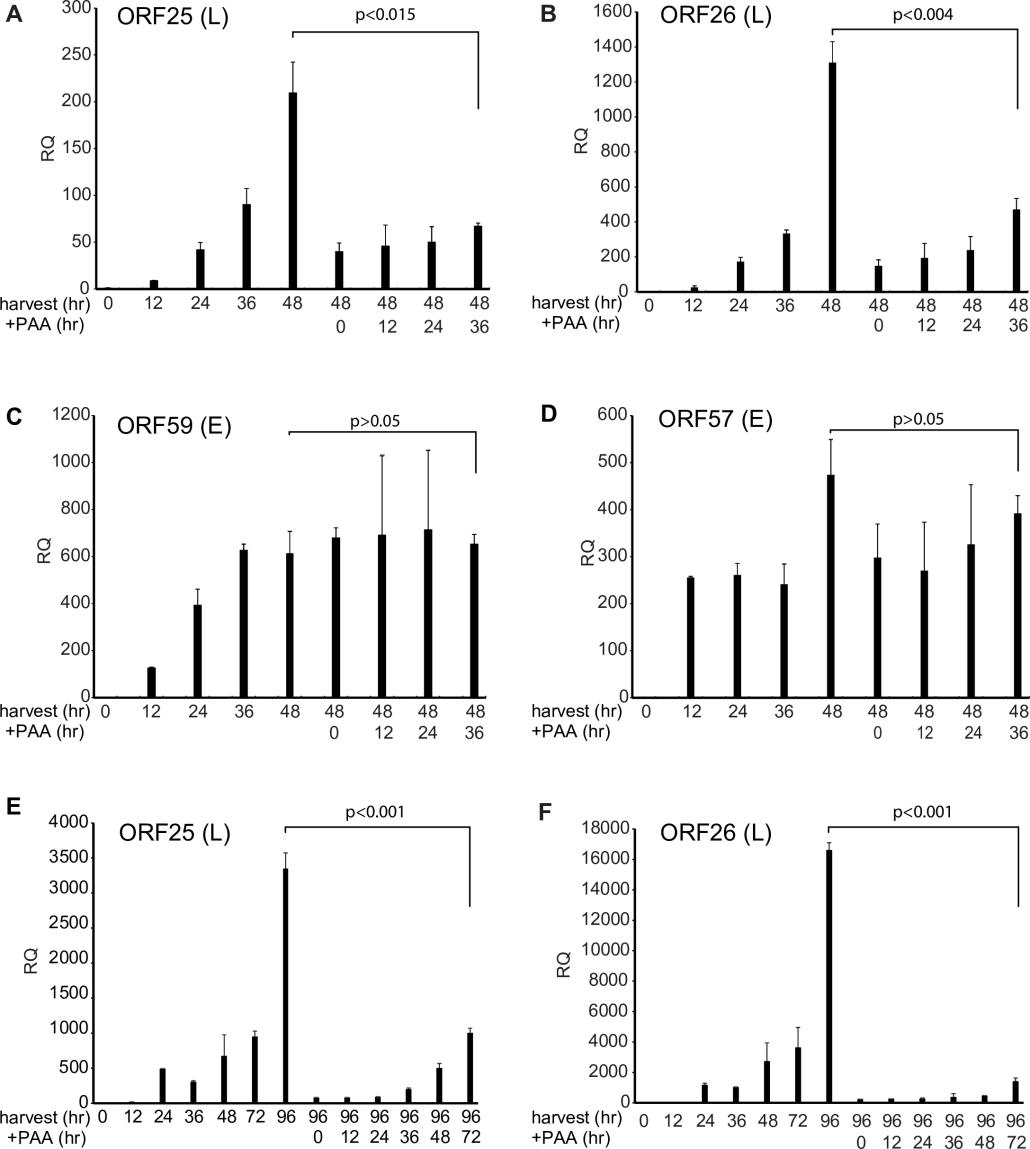
Effect on KSHV late mRNA accumulation of DNA replication blockade at various times after initiation of lytic replication. The effect of blocking DNA replication after induction on the subsequent accumulation of late gene mRNAs was assessed in KSHV infected cells. A-D. RNA accumulation at 48 hr post induction. iSLK cells were treated with doxycycline to induce lytic KSHV replication. RNA was harvested at different time points up to 48 hr as shown after induction at the left of each panel. DNA replication was also blocked at various times after induction (+PAA), RNA synthesis was allowed to proceed, and RNA was harvested at 48 hr, as shown at the right of each panel. qPCR was performed to measure relative quantity (RQ) of each lytic early gene [ORF59, ORF57] and late gene (ORF25, ORF26] as shown. E-F. RNA accumulation at 96 hr post induction. iSLK cells were treated with doxycycline and PAA as indicated. RNA was harvested at different time points up to 96 hr after induction (harvest), shown at the left of each panel. After blockade of DNA replication with PAA as in A-D above, RNA was harvested at 96 hr and qPCR was performed to measure relative quantity (RQ) of each lytic late gene (ORF25, ORF26]. Error bars represent SEM.

Since neither removal of DNA methylation nor increasing template number appeared to be the reason for the linkage of late gene expression to DNA replication, we considered the possibility that DNA replication removed other protein factors from latent episomes that are chromatinized during latency. If such a mechanism were involved, initiation of DNA replication could be sufficient to enable late gene transcription. Alternatively, if DNA replication were required to bring proteins required for late gene transcription to the late gene promoters, or were necessary to maintain a replication factory with the requisite transcription factors, continuing (ongoing) DNA replication would be necessary to maintain late gene transcription. To distinguish between these alternatives, we performed the following experiment to assess the effect of blocking DNA replication at various times after induction of lytic replication. We induced lytic KSHV replication in iSLK cells and added PAA at 0 hr, 12 hr, 24hr, 36hr post-induction, and then harvested RNA at 48 hr post induction. At each time point when we add PAA, we also harvested untreated cells in parallel to compare the temporal pattern of RNA accumulation. The results showed that KSHV late gene (ORF25 and ORF26) accumulation at 48 hr was inhibited compared to no PAA treatment when we added PAA at any time after induction (Fig 4A and 4B]. On the other hand, early gene (ORF59 and ORF57) accumulation did not change (Fig 4C and 4D). These data suggest that KSHV late gene expression requires ongoing DNA replication to maintain transcription. If initiation of DNA replication merely established a permissive condition for late gene transcription, accumulation should continue to increase even after PAA was added. We then further extended the time course to 96 hr post-induction and added PAA up to 72 hours post-induction to maximize the opportunity for genomes potentially licensed by DNA replication to transcribe late genes. As shown in Fig 4E and 4F, late gene RNA accumulation at 96 hr was still inhibited when we added PAA at any time point compared to no PAA treatment. Addition of PAA at any time point essentially led to cessation of further RNA accumulation. These results demonstrated that continued KSHV late gene mRNA accumulation requires maintaining ongoing viral DNA replication.

**Fig 5:**
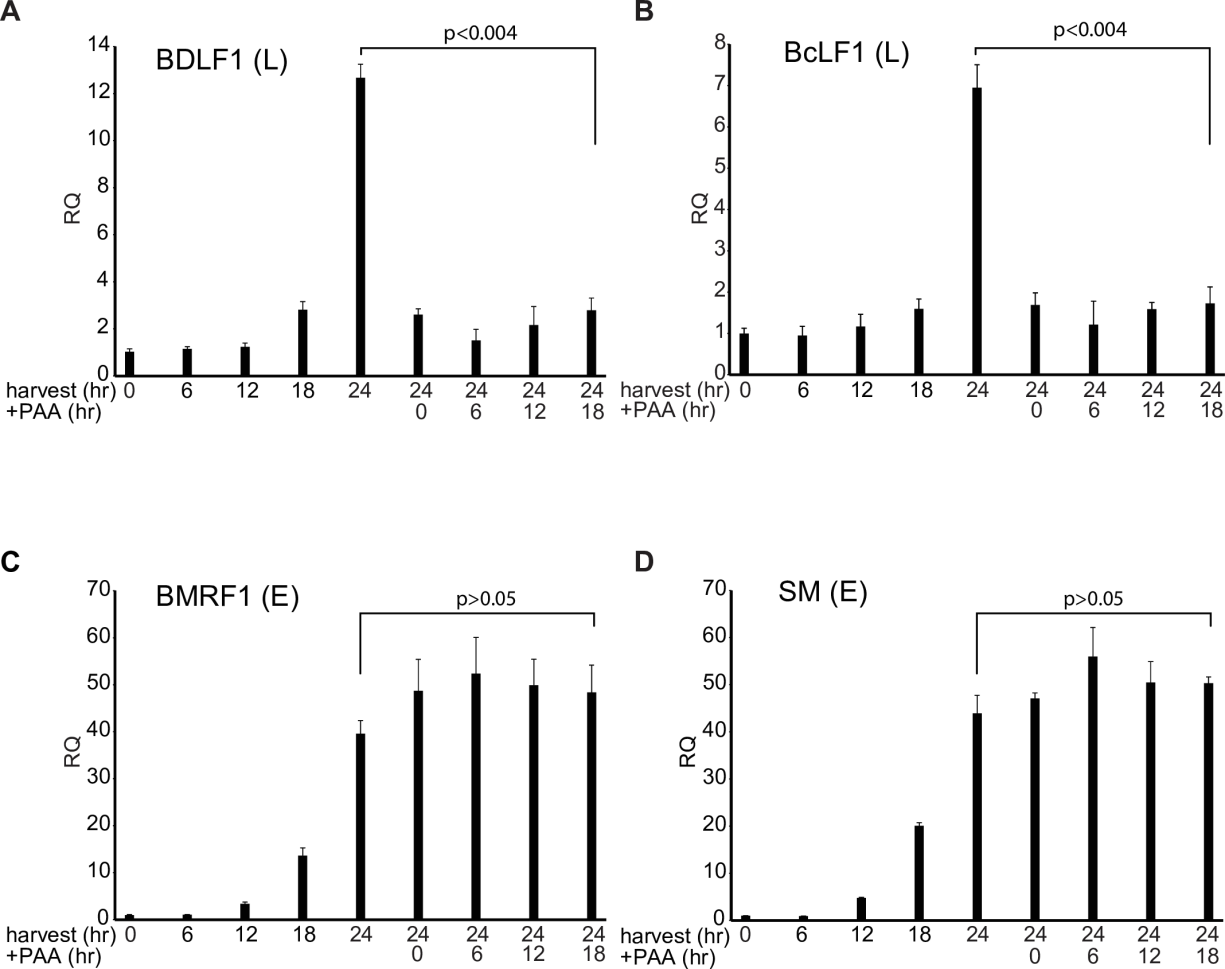
Effect of serial replication blockade on EBV lytic mRNA accumulation. The effect of blocking DNA replication after induction on the subsequent accumulation of late gene mRNAs was assessed in EBV infected cells. AGSiZ cells were doxycycline (+D) treated to induce lytic replication. RNA was harvested at different time points after induction up to 24 hr post-induction as shown at the left of each panel. PAA was added to block DNA replication at 0, 6, 12 and 18 hr, RNA synthesis was allowed to proceed, and RNA was harvested at 24 hr, as shown at the right of each panel. qPCR was performed to measure relative quantity (RQ) of each lytic early gene [BMRF1, SM] (C,D) and late gene (BDLF1, BcLF1] (A,B) as shown. Error bars represent SEM.

We next asked whether EBV late gene expression also required DNA replication for continued late mRNA transcription. We performed an analogous experiment with EBV in AGSiZ cells. Since EBV replication and increases in DNA copy number occur earlier post-induction in this system than in iSLK cells (Fig 2), we measured RNA accumulation at 24 hr post-induction and added PAA at serial times prior to 48 hr (0, 6, 12, and 18 hr). Similar to KSHV, EBV late gene (BDLF1 and BcRF1) mRNA accumulation at 24 hr was inhibited when we added PAA at any time point (Fig 5A and 5B) while early gene (BMRF1and SM) accumulation was unaffected (Fig 5C and 5D). These results confirmed that late gene transcription requires ongoing DNA replication in both KSHV and EBV.

### Inhibition of DNA replication causes dissolution of the EBV replication/transcription compartment

**Fig 6:**
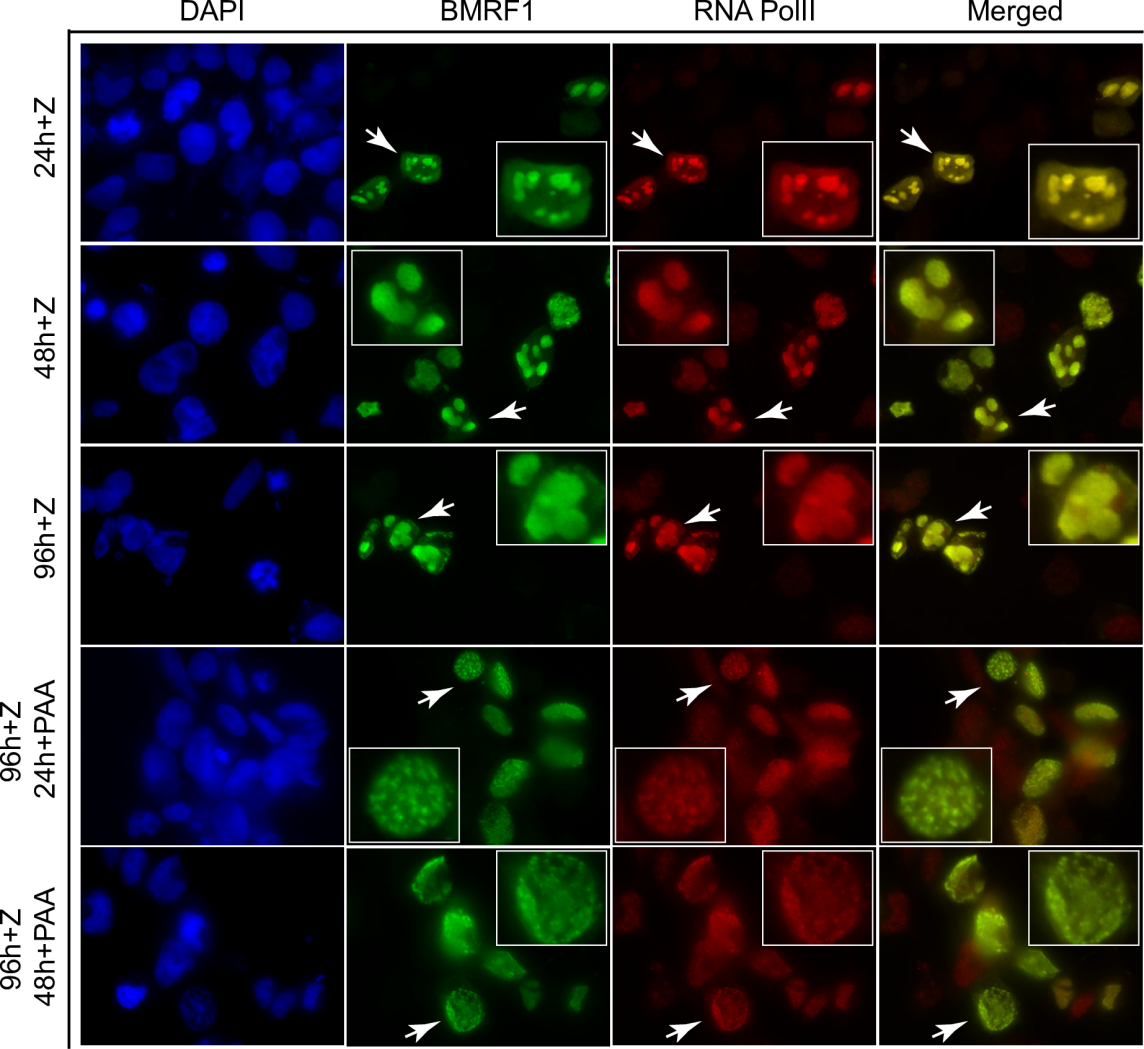
Dynamic changes of viral replication factories with PAA treatment. EBV B95-8 bacmid 2089 infected 293 cells were transfected with Zta expression vector (+Z) to induce EBV lytic replication and fixed at 24hr (24h + Z), 48 hr (48h + Z) or 96 hr (96h + Z) after transfection. Fixed cells were stained for RNA Pol II (Red) and BMRF1 (Green) to visualize the formation of replication compartments. Cells were also treated with PAA to block viral replication at 24 hr (24h + PAA) or 48 hr (48h +PAA) post-induction and fixed at 96 hr post-transfection to assess the effect of blocking DNA replication on replication compartment structure. Enlarged images of replication factories are shown in each panel and arrows indicate magnified cell. BMRF1, the polymerase-associated processivity factor was used as a marker for viral replication foci.

A potential explanation for the dependence of late gene transcription on continued DNA replication is that the process of lytic replication, which involves formation of nuclear replication factories, that include recruited transcription factors [23, 26], is essential for maintenance of the transcriptional milieu required for late gene transcription. We therefore wished to ask whether inhibiting DNA replication could affect the maintenance of EBV replication factories. The EBV DNA polymerase processivity factor BMRF1 (EA) concentrates in EBV replication factories and serves as a marker of these compartments in immunofluorescence studies [26, 36]. Identification and visualization of these nuclear replication compartments is easily performed in EBV infected 293 cells by staining for BMRF1. We first performed preliminary experiments in which we followed the formation of the replication compartments after induction of lytic replication. We induced EBV replication in EBV-infected 293 cells by transfection of lytic activator Zta expression plasmid. We then fixed and stained the cells with anti-BMRF1 antibody at various times post-induction. As shown in Fig 6, formation of replication factories is clearly evident by 24 hr post-induction, and continues through 96 hr. Consistent with previous reports [26], concentration and co-localization of RNA pol II in these nuclear replication foci is also evident (but not in cells in which EBV replication was not induced). We then asked what would happen to these replication compartments if DNA replication were blocked after they had been allowed to form. We added PAA after the formation of replication complexes, at 24 hr and 48 hr post replication induction, and stained the cells at 96 hr. Addition of PAA at either time point led to loss of the large, discrete foci typical of cells in which DNA replication was not blocked. Rather, BMRF1 became dispersed throughout the nucleus in fine speckles. RNA pol II remained associated with these residual speckles, also assuming a diffuse nuclear distribution. These data suggest that continuing DNA replication is required to maintain a nuclear replication/transcription factory.

**Fig 7:**
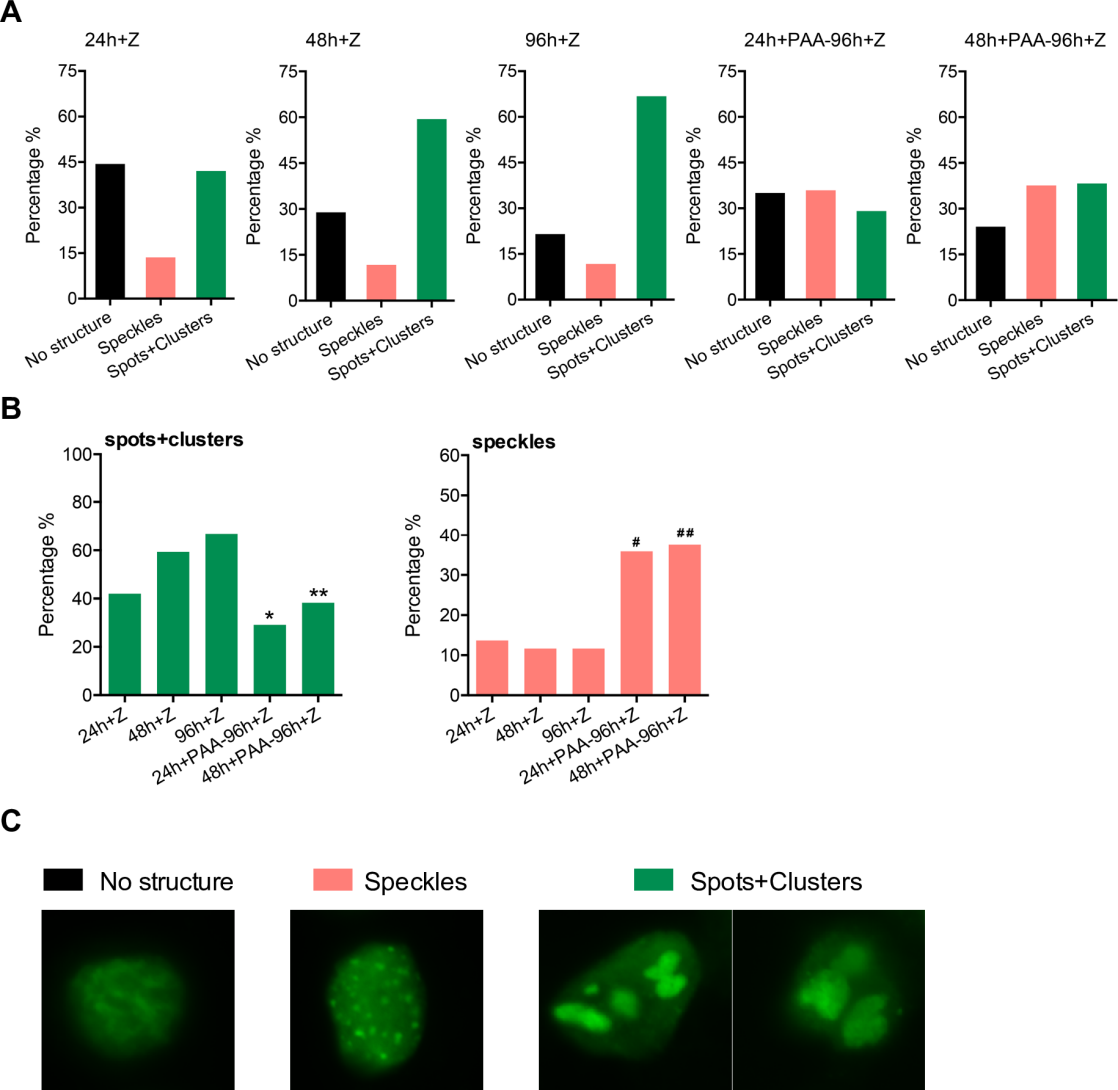
Kinetics and characteristics of viral replication factories after PAA treatment. Manual cell counting was performed to quantify the morphological changes of viral replication factories induced by inhibiting viral DNA replication. 15–20 fields of each slide were randomly chosen for counting under the objective magnification of 63X. A minimum of 150 cells were counted. Cell numbers were expressed as percentage of cells permissive of lytic replication (all BMRF1 positive cells). (A) Quantification of morphological changes of viral replication foci by manual cell counting. Each BMRF1 positive cell was categorized as either having no structure, speckles or spots/clusters. Characteristics of each type of structure are shown in micrographs of representative cells in (C) below. BMRF1, the polymerase-associated processivity factor (stained green) was used as a marker for viral replication foci. (B) Statistical analysis of changes in both large foci (spots and clusters) and small foci (speckles) under indicated conditions. * significant difference from PAA-untreated samples at 24 hrs (p<0.05) and at 96 hrs (p<0.001); ** significant difference from PAA-untreated samples at 48 hrs (p<0.001) and at 96 hrs (p<0.0001); # significant difference from PAA-untreated samples at 24 hrs (p<0.0001) and at 96 hrs (p<0.001); # # significant difference from PAA-untreated samples at 48 hrs and at 96 hrs (p<0.0001).

In order to confirm the observations shown above, we measured the changes in replication complex structure by counting the percentage of cells that contained the large clusters and smaller clusters (spots) versus the fine speckles more commonly seen after PAA treatment. As shown in Fig 7A, the number of cells with clearly formed larger replication foci (spots/clusters) increased over time with over 65% of BMRF1-positive cells containing such complexes. However, when PAA was added at 24 or 48 hr, by 96 hr, the number of cells with large foci had decreased to 29% and 38% respectively. While cells with replication foci do not completely disappear upon replication inhibition, they undergo decreases that are highly significant when compared to their initial prevalence at the time of PAA addition and at 96 hr (Fig 7B, left panel). As expected, the percentage of cells with speckles increased commensurately after PAA addition as shown in Fig 7B (right panel). Representative cells of each category are shown in Fig 7C.

**Fig 8:**
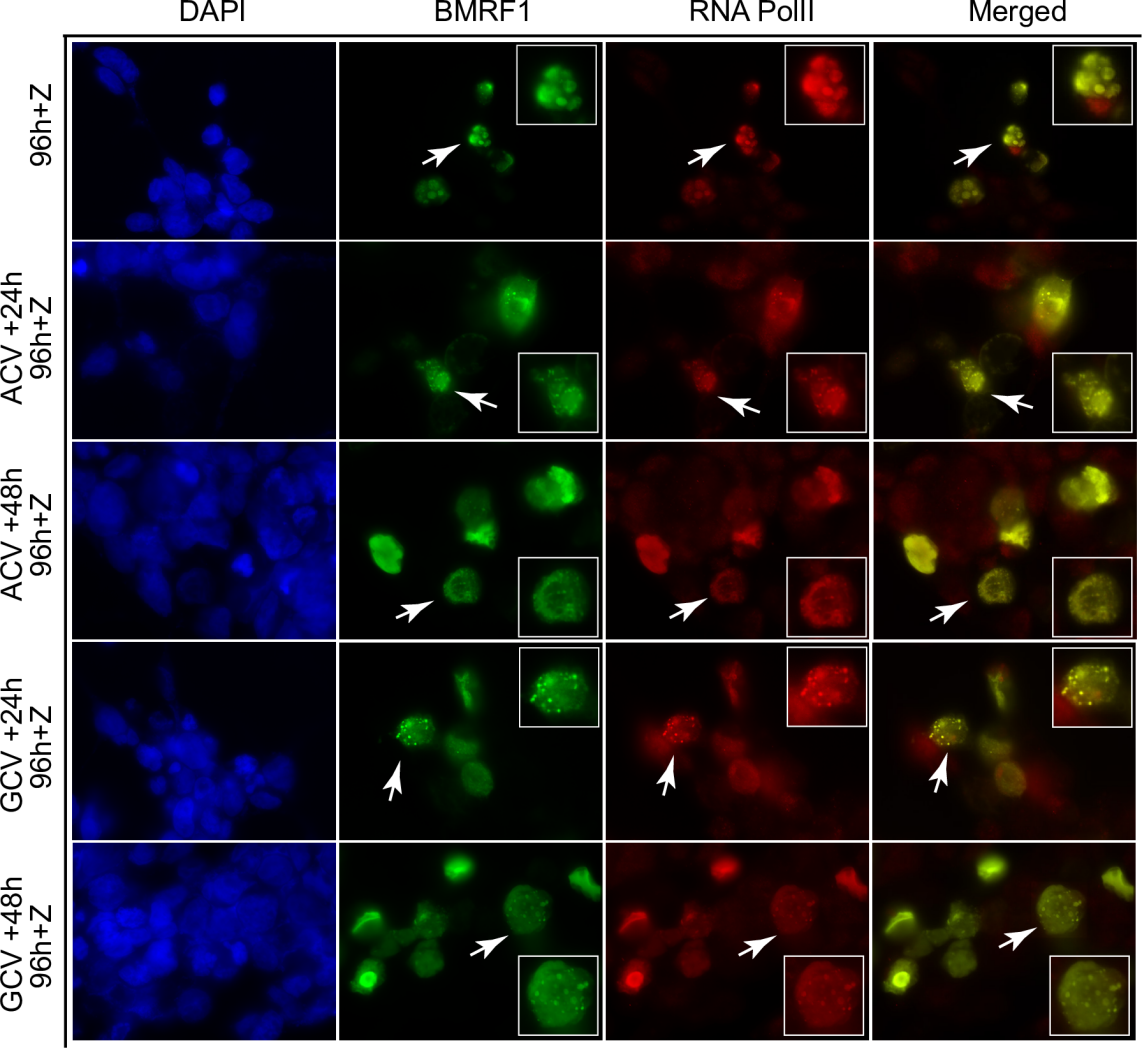
Dynamic changes of viral replication factories with ACV or GCV treatment. EBV B95-8 bacmid 2089 infected 293 cells were transfected with Zta expression vector (+Z) to induce EBV lytic replication and mock-treated or treated with ACV (acyclovir, 25 μg/ml) or GCV (ganciclovir, 5 μg/ml) to block viral replication at 24 hr or 48 hr post-induction. Both treated and untreated cells were stained for RNA Pol II (Red) and BMRF1 (Green) at 96 hr post transfection. Arrows indicate magnified cells in each panel.

In order to confirm that these effects were not unique to PAA, we used two additional inhibitors of EBV DNA replication, acyclovir and ganciclovir to assess their effect on replication complex maintenance. AGSiZ/EBV cells were induced to permit EBV replication and the replication inhibitor was added either 24 or 48 hr later and cells were fixed at 96 hr post-induction. Cells untreated with either drug were induced and fixed at 96 hr post induction exactly as performed previously with PAA. Fixed cells were then stained with anti-BMRF1 and anti-RNAP antibodies and examined by fluorescence microscopy. The results, shown in Fig 8, confirm that inhibiting EBV DNA replication with either ganciclovir or acyclovir leads to loss of replication complex integrity. As with PAA, RNA pol II appears to maintain association with BMRF1 in the finer speckles resulting from blocking DNA replication.

### Inhibition of DNA replication leads to dissociation of an EBV protein required for late gene transcription from replication factories

**Fig 9:**
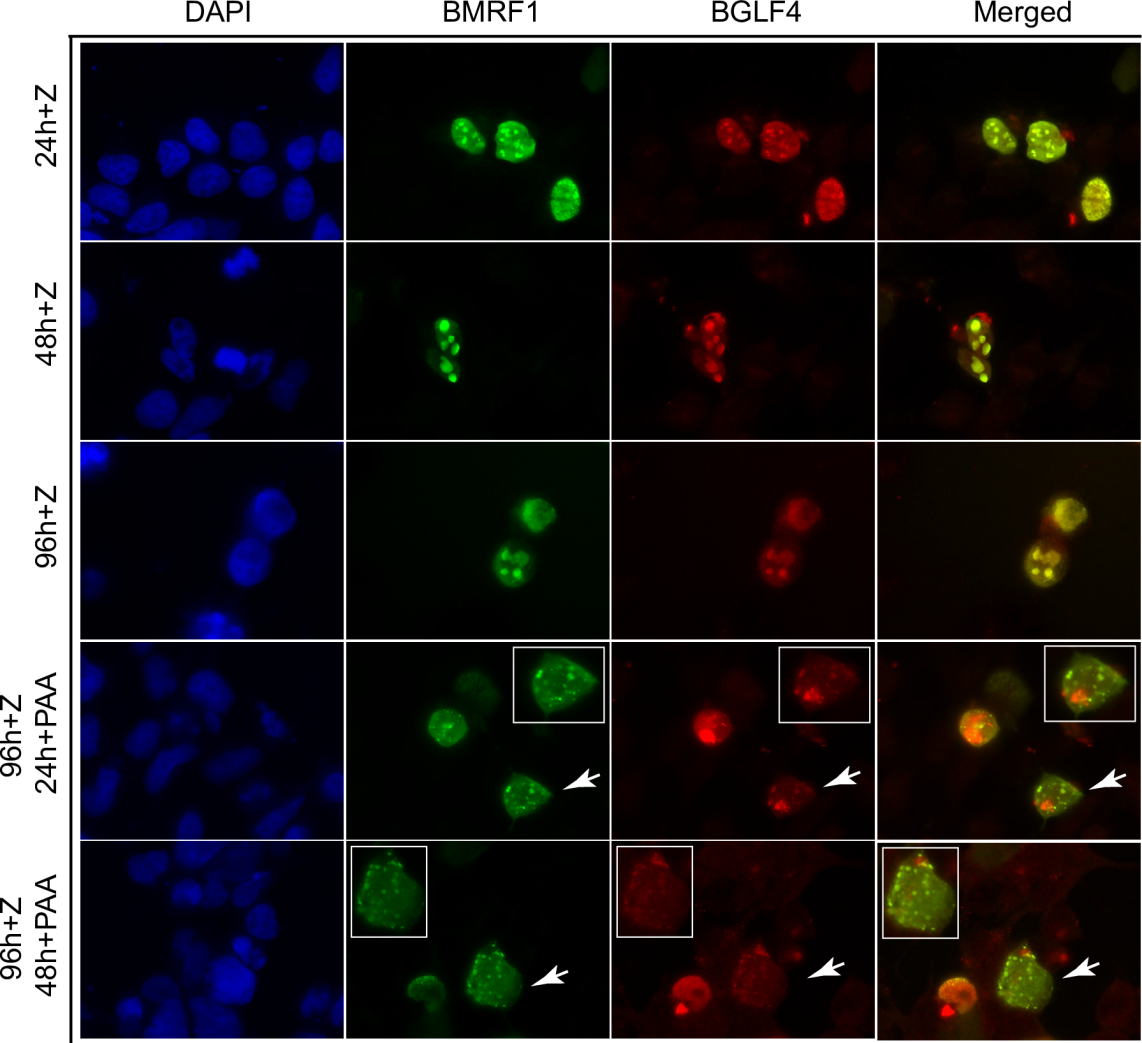
Effect of inhibiting viral DNA replication on co-localization of BMRF1 and BGLF4 during EBV lytic replication. EBV B95-8 bacmid 2089 infected 293 cells were transfected with Zta expression vector (+Z) to induce EBV lytic replication and mock-treated or treated with PAA to block viral replication after replicative complex formation, at 24 hr or 48 hr post-induction. Cells were then stained for BGLF4 (Red) and BMRF1 (Green) at the indicated times post induction. All PAA treated cells were fixed and stained at 96 hr post-induction. BMRF1, the polymerase-associated processivity factor was used as a marker for viral replication foci.

**Fig 10:**
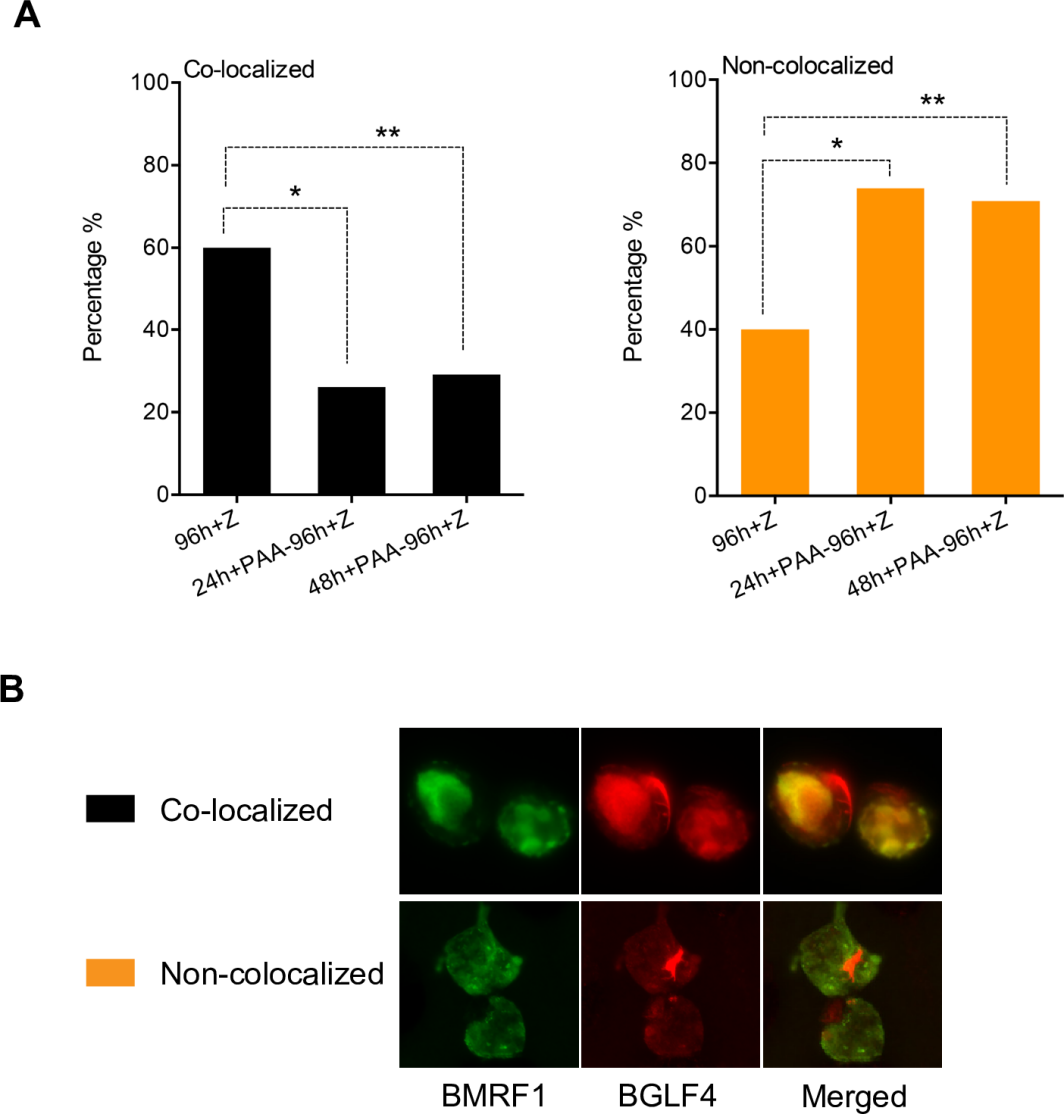
Measurement and analysis of BMRF1/BGLF4 nuclear co-localization after PAA treatment. Manual cell counting was performed to quantify the morphological changes of viral replication factories. 15–20 fields of slides were randomly chosen for counting under the objective magnification of 63X. Cell numbers were expressed as percentage of replicating cells (BMRF1 positive cells). Each cell was categorized as either demonstrating colocalization if greater than 75% of the BMRF1 and BGLF4 signal was overlapping. (A) Percentage changes in co-localization between BMRF1 and BGLF4 after PAA treatment at either 24 or 48 hr post-induction were calculated. * p value < 0.0001, ** p value <0.001. (B) Representative cells categorized as either colocalized or non-colocalized are shown.

Disintegration of replication complexes upon blockade of DNA replication suggested that complex integrity might be necessary for recruitment of transcription factors. Since RNA pol II appeared to remain associated with BMRF1 even after complex dispersal, we wished to ask whether inhibition of DNA replication might lead to loss of other factors needed for late gene transcription. BGLF4, an EBV encoded kinase with multiple functions in viral packaging and egress [37, 38], has also recently been shown to be essential for transcription of several late genes [39] and to be present in replication complexes [40]. We therefore examined the location of BGLF4 after inducing EBV replication and after inhibiting DNA replication. In cells undergoing replication, BMRF1 was localized in mostly large structures. BGLF4 was found in these structures as well, although the co-localization with BMRF1 was not 100%. Whereas the majority of BMRF1 complexes contained BGLF4, nuclear foci of BGLF4 without BMRF1 were also present (Fig 9, 48h + Z). After treatment with PAA, the BMRF1 foci exhibited dispersal as expected. In addition, BGLF4 was no longer highly co-localized with BMRF1. In order to quantify and confirm this effect, we counted nuclei that were BMRF1 positive and categorized the nuclei as either co-localized or not co-localized. Cells where greater than 75% of the BMRF1 foci also contained BGLF4 were counted as co-localized and vice versa (Fig 10). The number of cells exhibiting co-localization decreased significantly after PAA treatment. These data indicate that when the replication factories undergo dispersal, there is a concomitant loss of BGLF4 recruitment.

## Discussion

We have investigated the factors that link late gene transcription to lytic DNA replication in the γ herpesviruses EBV and KSHV. We have shown the DNA demethylation that occurs consequent to lytic replication of KSHV and EBV genome by viral DNA polymerases is unlikely to be the mechanism by which late gene transcription is linked to DNA replication *in cis*. Increases in late gene expression also preceded increases in DNA template copy number, confirming that template amplification does not contribute significantly to late transcript accumulation. Temporal analysis of the relationship between DNA replication blockade and late mRNA accumulation indicated that KSHV and EBV late gene expression require ongoing DNA replication. Further, RNA pol II and the EBV kinase BGLF4 colocalized with DNA polymerase processivity factor in EBV replication factories and blockade of DNA replication led to loss of replication compartment integrity.

DNA methylation has an important regulatory role in gene expression during the KSHV and EBV life cycle. The EBV and KSHV genomes are highly methylated during latency, and become hypomethylated or unmethylated during lytic infection [29, 41]. Unlike cellular DNA or latent herpesvirus genomes, newly produced herpesviral genomes are not remethylated after lytic replication, and virion DNA is unmethylated [42–44]. DNA demethylation has been shown to upregulate viral gene expression and induce EBV and KSHV lytic replication [29, 41, 45]. Thus, it was plausible that replication-dependent demethylation was required for late gene promoter activity. We found that viral DNA demethylation by 5-Aza treatment potentiated both EBV and KSHV early and late lytic gene expression (Figs 2, 3 and 4D). Although demethylation increased both early and late lytic promoter activity, it could not rescue late gene transcription from DNA replication blockade. These data therefore indicate that removal of CpG promoter methylation is not the mechanism of late gene expression dependence on DNA replication.

Previous studies using plasmids with lytic herpesviral origins and late gene promoters have suggested that increases in DNA template number are not required to support late gene expression *in cis* [11]. In order to confirm these findings in the context of entire viral genomes, we measured the viral DNA content of lytically induced KSHV and EBV infected cells over time and compared it to the temporal accumulation of late gene transcripts. The increase in transcript levels occurred prior to increases in DNA copy number, confirming that replication induced template abundance does not play a significant role in enhancing late gene transcription. Thus, DNA replication has an effect on late gene transcription very early after it begins and before template number is significantly increased.

It therefore appeared that late gene transcription occurs from newly replicated genomes, and some property of the newly replicated DNA makes it supportive of late gene transcription. Two alternative models for the permissiveness of nascent herpesvirus genomes can be envisioned with slightly different implications. First, a topological or steric property of newly replicated DNA allows access of the late gene vPIC and the RNA polymerase to the relevant promoters. Such a mechanism would suggest that once a linear or partly linear, non-supercoiled genome were produced, it would be permissive for continued late gene transcription, and subsequent blockade of DNA replication would not shut off persistent late gene expression from the “licensed” genomes. Alternatively, a mechanism that requires continuing operation of the DNA polymerase might be required. The DNA polymerase or associated factors might bring or recruit essential transcription cofactors to the late gene promoters as has been demonstrated to occur during replication of bacteriophage T4 [46]. A recent report of interaction between ORF59, the KSHV DNA polymerase processivity factor and RNA pol II make this an attractive hypothesis [47]. In this alternative scenario, blocking DNA replication at any point would strongly inhibit, if not curtail, further late gene expression as continued delivery of proteins essential for transcription would cease. A related model is one in which the replication factory: a complex of the vPIC, late gene transcription factors, RNAP, the replicating genome and DNA polymerase, requires ongoing DNA replication to be maintained. Finally, if linear genomes enter a non-permissive state for late gene transcription shortly after replication, either by temporary chromatinization or encapsidation, continued production of new templates would be required.

We first asked whether DNA replication licensed templates for continued late gene transcription, that is, whether stopping DNA replication would still allow late gene transcription from linear genomes that had previously been synthesized. We inhibited DNA replication at various times after induction of lytic transcription, and either harvested RNA or allowed potential transcription to proceed and measured RNA accumulation at the end of a fixed period allowed for transcription. A similar analysis of HSV late gene transcription concluded that continued DNA replication was not required for additional synthesis of late gene mRNAs [48]. However, in the case of both KSHV and EBV, we found that inhibiting DNA replication essentially led to a cessation of further RNA accumulation beyond that point.

**Fig 11:**
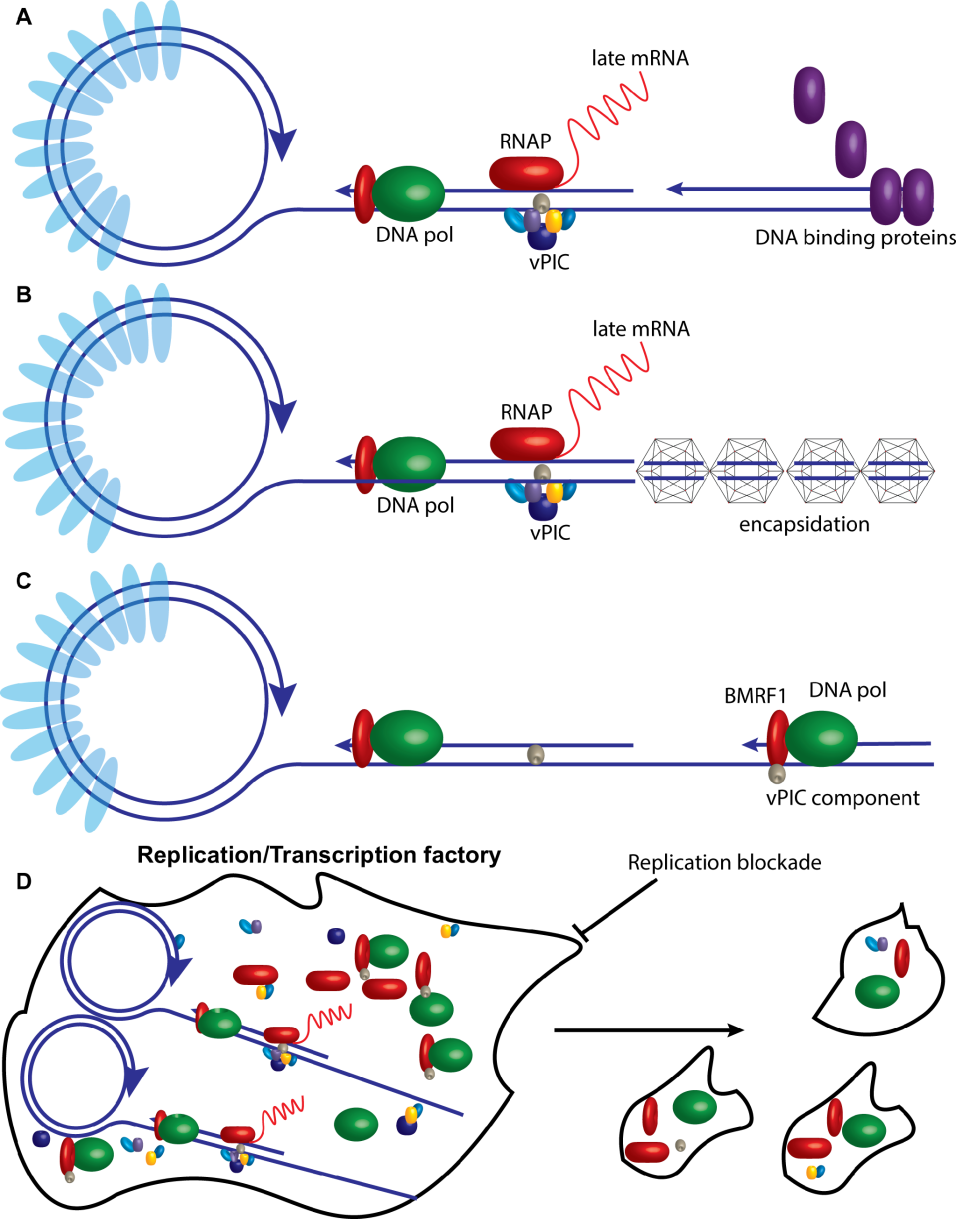
Models for the dependence of late gene transcription on continuing DNA replication. A. Newly replicated linear herpesvirus genomes are shown being bound by DNA binding proteins shortly after production, rendering them inaccessible for the late gene transcription complex. Histones bound to latent genomes prior to lytic transcription are depicted in light blue. Only newly replicated genomes devoid of protein are shown being transcribed. B. Replicated genomes are shown being encapsidated, preventing further transcription which only occurs from newly produced genomes. C. The translocating DNA polymerase complex is shown bringing a component of the vPIC to a late gene promoter. BMRF1 is shown as the protein performing this function by analogy to T4 replication, but the process may be performed by any component of the DNA polymerase machinery, and one or more proteins required for late gene transcription may be involved. D. The replication/transcription factory is shown undergoing dissolution when the process of DNA replication is blocked, with a loss of physical integrity and dispersion of the locally concentrated replication proteins, DNA template and RNA transcription factors. Some of the interacting proteins are shown as maintaining association in the dispersed particles.

Thus, it appears that in the absence of continuing DNA replication, the newly replicated genomes do not remain competent for transcription. Possible explanations for this phenomenon stem from the basic concept that changes occur to the nascent genomes shortly after their production that render them no longer accessible for transcription. One mechanism by which this could occur is the deposition of proteins on linear genomes that prevent further transcription unless removed by additional rounds of replication as depicted in Fig 11A. While it is generally accepted that herpes virus genomes prior to packaging are devoid of histones or other chromatin marks, it is possible that linear genomes undergo a period of rechromatinization prior to the removal of histones upon encapsidation. However, histones have been reported to be excluded from EBV replication factories during late stages of lytic replication [49]. Similarly, a recent report shows that newly replicated KSHV genomes do not associate with histones [50]. Thus, we consider this model less likely as an explanation of DNA replication and late transcription linkage. Nevertheless, it remains possible that other proteins may bind to the newly replicated genomes after synthesis [50], and such proteins may prevent continued access of the late gene transcription complex to the DNA.

An alternative model is that topological changes in the newly replicated genomes allow access to late gene transcription complexes. There may be a limited period of time shortly after synthesis when the linear genomes sterically allow access of the RNA polymerase complex but then assume conformations, perhaps due to encapsidation, that render them no longer accessible to the RNA polymerase machinery (Fig 11B).

Finally, replication may also be necessary to continually bring or recruit factors necessary for late gene transcription to the late gene promoters (Fig 11C). A distinct, although not mutually exclusive, mechanism is suggested by our finding that there is a physical dissolution of the replication complexes upon cessation of EBV DNA replication. The process of ongoing DNA replication thus seems to be required to maintain the integrity of the replication factory. Consistent with previous findings, we have shown that RNA pol II co-localizes with BMRF1 during EBV lytic replication in large nuclear structures [26]. The late gene vPIC complex is recruited to noncanonical TATT boxes of true late gene promoters; components of the vPIC have been shown to interact with RNA polymerase II; and late gene mRNAs localize to the inner cores of replication factories [8, 18, 26, 51]. BGLF4, which is required for transcription of late genes encoding structural proteins [39], also is found in the replication complex [52]. The fact that these replication/transcription factories, in which late lytic gene transcription and DNA replication occur, undergo breakdown when DNA replication stops, suggests that a functioning complex of DNA polymerase and viral genome is required to maintain the physical and functional integrity of the structure. When DNA replication stops, the replication/transcription factory begins to disintegrate (Fig 11D). It appears that while some association of DNA replication proteins with transcription machinery, such as with RNAP, may persist, others such as with BGLF4, may not. Ultimately it appears that late gene transcription is dependent on a on a very tight physical and temporal association with ongoing lytic DNA replication. Such a mechanism would help to ensure that late gene products are synthesized concurrently and at the appropriate time to be available for packaging of newly replicated genomes.

## Materials and methods

### Cells and plasmids

iSLK cells [32], kind gift of Don Ganem, were maintained in DMEM containing 10% charcoal stripped FBS (Sigma) and 1% glutamine with 250 μg/ml G-418 and 1 μg/ml puromycin. iSLK cells were infected with WT KSHV derived from bacmid BAC16, expressing eGFP and hygromycin resistance (kind gift of Jae Jung) [31]. KSHV infected iSLK cells were maintained in 1.2 mg/ml hygromycin, 250 μg/ml G-418 and 1 μg/ml puromycin. We generated an EBV positive gastric carcinoma cell line AGSiZ by transducing AGS/BX1 cells [53](gift of Lindsey Hutt-Fletcher), with a lentivirus expressing the EBV transactivator protein Zta (BZLF1) under control of a doxycycline inducible promoter [34, 54]. Cells were grown in F-12 media containing 10% charcoal stripped FBS (Clontech) and 1% glutamine with 500 μg/mL G-418 and 0.5 μg/mL puromycin. For demethylation of iSLK cells or AGSiZ cells, iSLK or AGSiZ cells were cultivated in media supplemented with 2 μM 5-Aza for two days. Viral lytic replication was induced by treatment with 1 μg/ml doxycycline. For inhibition of viral DNA replication, 500nM PAA was added simultaneously with 5-Aza. 293 cells infected with 2089 B95-8 bacmid (kind gift of Henri-Jacques Delecluse) [55] were grown in DMEM with 10% FBS and 100 μg/ml hygromycin.

## DNA and RNA isolation and analysis

To induce KSHV or EBV lytic gene expression and virus replication, iSLK or AGSiZ cells were treated with 1 μg/ml doxycycline. Cells were harvested at different times as indicated. Total cellular DNA or RNA was isolated from washed cell pellets using Qiagen DNeasy Blood & Tissue Kit (for DNA) or Qiazol and Qiagen miRNeasy columns (for RNA) according to the manufacturer’s protocols. All experiments were performed in triplicate with three biological replicates. Real-time quantitative PCR (qPCR) for DNA was performed with SYBR green PCR Master Mix (Applied Biosystems) according to the manufacturer’s protocol. Reverse transcribed real-time quantitative PCR (RT-qPCR) was performed with the Power SYBR Green RNA-to-C_T_ 1-Step kit. Each biological replicate sample was analyzed in triplicate with gene specific primers and β-actin was used as the endogenous control. The gene-specific primers were as follows:

K8.1F: 5’- GGT CGC AGA AAA CGG CAG AAA TAG-3’

K8.1 R: 5’-AAG TCC CAG CAA TAA ACC CAC AGC-3’

ORF6-2093F: 5’-CTGCCATAGGAGGGATGTTTG-3’

ORF6-2158R: 5’-CCATGAGCATTGCTCTGGCT-3’

ORF25-3733F:5’-CTCGGCGACGTGCTATACAAT-3’

ORF25-3803R: 5’-TGCCGACAAGGACTGTACATG-3’

ORF26 FLORE 1F: 5’-AGCCGAAAGGATTCCACCAT-3’

ORF26 FLORE 1R: 5’-TCCGTGTTGTCTACGTCCAG-3’

ORF57 Q1-5: 5’-GCAGAACAACACGGGGCGGA-3’

ORF57Q2-3: 5’-GTCGTCGAAGCGGGGGCTCT-3’

ORF59 Q1F, 5’-CTCCCTCGGCAGACACAGAT-3’;

ORF59 Q1R, 5’-GCGTGGTGCACACCGACGCCC-3’;

BcLF1 Q1F: 5’-GTGGATCAGGCCGTTATTGA-3’

BcLF1 Q1R: 5’- CCTCAAACCCGTGGATCATA-3’

BCRF1 Q1F: 5’- GACAAAGGACGAGGTAGATAA-3’

BCRF1 Q1R: 5’-CTCCAGGTAGAATTGGATCATT-3’

BDLF1 Q1F: 5’-TGGATGAGGTTAGCGTGGACAGTT-3’

BDLF1 Q1R: 5’-TCTAACTTCACGGTGGCATGCTCT-3’

BMRF1 Q1F: 5’-ATACGGTCAGTCCATCTCCT-3’

BMRF1 Q1R: 5’-CACTTTCTTGGGGTGCTT-3’

SM Q1F: 5’-GGGCTGGGCAAGGTGACAAAT-3’

SM Q1R: 5’- AGGAAGCAGGCGAGGCAAGAA-3’

β-actin Q1F: 5’-TCAAGATCATTGCTCCTCCTGAG-3’

β-actin Q1R: 5’-ACATCTGCTGGAAGGTGGACA-3’;

### Bisulfite conversion and COBRA assays

800 ng genomic DNA was treated with sodium bisulfite using an EpiTech Bisulfite Kit (Qiagen) according to the manufacturer’s protocol. Two microliters of bisulfite converted DNA were subjected to 39 cycles of PCR with the following primers to amplify the PKP4 locus [35]:

PKP4 1F: GGTATGATTTTAAAAAAAGAGAT

PKP4 1R: GTAAAACCCTCCGAACCAAATATAAA.

PKP4 PCR product (3ul) was digested with TaqI (NEB) at 65°C for 2.5 hrs. The undigested and digested samples were separated in a 2% agarose/ethidium bromide gel to assess the demethylation effect of 5-Aza. Cleavage only occurs if the cytosines in the restriction sites are preserved as cytosines during the bisulfite modification as a result of methylation.

### Pyrosequencing

Methylation status of CpG sites in the KSHV ORF33 promoter region was examined by a pyrosequencing-based methylation assay using the PyroMark Q24 instrument (Qiagen). Briefly, primers for the polymerase chain reaction (PCR) and pyrosequencing were designed using PyroMark Assay Design Software 2.0 (Qiagen). Bisulfite-converted DNA samples were amplified using the PyroMark PCR kit (Qiagen) with following PCR primer sets:

ORF33 BS1F: TGTTGGATGGAGGTGTTAGGATTATG

ORF33 BS1R: ACCTTTAATAACAAAACCCCCAAAT (Biotinylated).

ORF33 seq1: GTGTTAGGATTATGGGAAA was used for pyrosequencing in the PyroMark Q24 instrument. Data analysis was carried out with PyroMark Q24 Software (Qiagen).

### Immunofluorescence microscopy

293 cells infected with 2089 B95-8 bacmid [55] were grown on glass coverslips plated at 250,000 cells per well in six-well dishes and transfected with Zta expression plasmid (+Z) to induce EBV lytic replication by using TransIT293 (Mirus) according to the manufacturer’s protocol. PAA (500nM final concentration) was added to block viral replication at different time points as indicated in the text. 96 hours after transfection, cells were washed with 1x PBS, fixed and permeabilized with PBS containing 4% paraformaldehyde and 0.2% Triton X-100 for 15–20 minutes at room temperature, and then washed two times with 1x PBS followed by incubating with blocking buffer (20% goat serum in PBS) for 30 mins at room temperature. Finally, the cells were incubated with anti-BMRF1 monoclonal antibody (Capricorn) and polyclonal anti-RNAP antibody at 37°C for 1 hour. For BGLF4 visualization, rabbit anti-BGLF4 antibody, kind gift of Ayman El-Guindy [39], was used at a dilution of 1:1000. The slides were washed three times with 1x PBS and incubated with secondary antibody Alexa Fluor 594 goat anti-mouse IgG, or Alexa Fluor 647 goat anti-rabbit IgG for 1 hour at 37°C (in the dark). Nuclear staining was performed with 4’,6- diamidino-2-phenylindole (DAPI) (Invitrogen). Images were collected and analyzed with a ZEISS Imager M2 microscope system. Statistical testing for comparison of proportions and p values was performed using MedCalc software, which uses the "N-1" Chi-squared test [56].

## Supporting information

S1 Fig Pyrosequencing of the ORF33 promoter. The sequence from the ORF33 promoter to be analyzed (after bisulfite conversion) is shown above the chromatogram. Cells were either mock induced (-Dox) or induced with doxycycline (+Dox) and treated with either PAA or 5Aza as shown above each panel. Percentage methylation at each CpG site is shown above each highlighted base. Dispensation order is shown below.
(TIF)
