# Supplementary figures and images for: Continuous DNA replication is required for late gene transcription and maintenance of replication compartments in gammaherpesviruses
Source: PLoS Pathog. 2018 May 29;14(5):e1007070. doi: 10.1371/journal.ppat.1007070 (PMC5993329; doi:10.1371/journal.ppat.1007070)

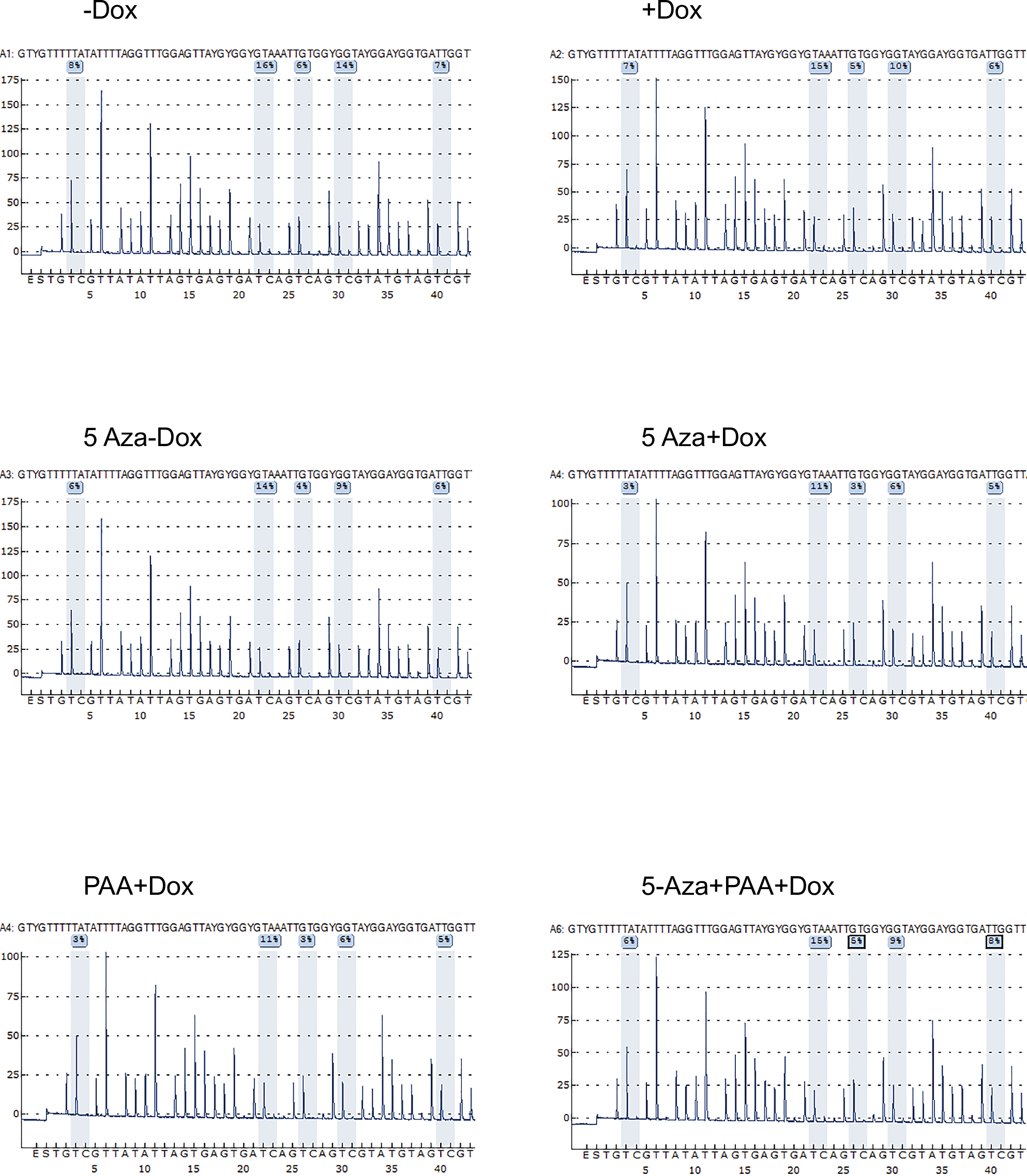

Supplement: S1 Fig — The sequence from the ORF33 promoter to be analyzed (after bisulfite conversion) is shown above the chromatogram. Cells were either mock induced (-Dox) or induced with doxycycline (+Dox) and treated with either PAA or 5Aza as shown above each panel. Percentage methylation at each CpG site is shown above each highlighted base. Dispensation order is shown below. (TIF) [file ppat.1007070.s001.tif]
